# Non-invasive Neuromodulation of Spinal Cord Restores Lower Urinary Tract Function After Paralysis

**DOI:** 10.3389/fnins.2018.00432

**Published:** 2018-06-29

**Authors:** Parag N. Gad, Evgeniy Kreydin, Hui Zhong, Kyle Latack, V. Reggie Edgerton

**Affiliations:** ^1^Department of Integrative Biology and Physiology, University of California, Los Angeles, Los Angeles, CA, United States; ^2^Rancho Los Amigos National Rehabilitation Center, Downey, CA, United States; ^3^Institute of Urology, Keck School of Medicine of University of Southern California, Los Angeles, CA, United States; ^4^Department of Neurobiology, University of California, Los Angeles, Los Angeles, CA, United States; ^5^Department of Neurosurgery, University of California, Los Angeles, Los Angeles, CA, United States; ^6^Brain Research Institute, University of California, Los Angeles, Los Angeles, CA, United States; ^7^Institut Guttmann, Hospital de Neurorehabilitació, Institut Universitari Adscrit a la Universitat Autònoma de Barcelona, Barcelona, Spain; ^8^Faculty of Science, The Centre for Neuroscience and Regenerative Medicine, University of Technology Sydney, Ultimo, NSW, Australia

**Keywords:** Non-invasive spinal cord stimulation, spinal cord injury, lower urinary tract, urodynamics, bladder function, paralysis

## Abstract

It is commonly assumed that restoration of locomotion is the ultimate goal after spinal cord injury (SCI). However, lower urinary tract (LUT) dysfunction is universal among SCI patients and significantly impacts their health and quality of life. Micturition is a neurologically complex behavior that depends on intact sensory and motor innervation. SCI disrupts both motor and sensory function and leads to marked abnormalities in urine storage and emptying. Current therapies for LUT dysfunction after SCI focus on preventing complications and managing symptoms rather than restoring function. In this study, we demonstrate that Transcutaneous Electrical Spinal Stimulation for LUT functional Augmentation (TESSLA), a non-invasive neuromodulatory technique, can reengage the spinal circuits' active in LUT function and normalize bladder and urethral sphincter function in individuals with SCI. Specifically, TESSLA reduced detrusor overactivity (DO), decreased detrusor-sphincter dyssynergia (DSD), increased bladder capacity and enabled voiding. TESSLA may represent a novel approach to transform the intrinsic spinal networks to a more functionally physiological state. Each of these features has significant clinical implications. Improvement and restoration of LUT function after SCI stand to significantly benefit patients by improving their quality of life and reducing the risk of incontinence, kidney injury and urinary tract infection, all the while lowering healthcare costs.

## Introduction

It is generally perceived that paralysis caused by spinal cord injury (SCI) only impacts one's ability to ambulate. However, restoration of autonomic functions such as bladder control are of the highest priority to SCI individuals (Anderson, 2004; Snoek et al., 2004). The function of the lower urinary tract (LUT) includes storage of urine without leakage and timely emptying without urine retention. All of the urinary problems encountered after a SCI are manifestations of impairments in these two functions of the LUT (Burns et al., 2001). The LUT is innervated by autonomic and somatic motor nervous system: parasympathetic fibers, originating from the parasympathetic nucleus (S2–S4) promote bladder contraction and voiding; sympathetic fibers originating from the thoracolumbar portion of the sympathetic chain promote bladder relaxation and bladder neck contraction, thus promoting continence; and somatic innervation arising from a distinct region of S2–S4 drives contraction of the external urethral sphincter (EUS), thus also promoting continence (de Groat and Yoshimura, 2001). In a healthy state, bladder and EUS activity are coordinated, with the EUS contracting and the bladder relaxing during urine storage, and the reverse occurring during voiding. Coordination of EUS-bladder activity is mediated by several nuclei in the brainstem. Because SCI disrupts communication between the brainstem and the lumbosacral cord, the bladder, and the EUS become uncoordinated, a condition known as detrusor-sphincter dyssynergia (DSD). The sensory innervations to the LUT are as important as motor fibers. The LUT transmits a variety of sensory information to the central nervous system and that transmission is also interrupted by SCI. As a result, myelinated Aδ fibers that normally ensure normal sensation are replaced by unmyelinated C fibers (de Groat, 1997; de Groat and Yoshimura, 2010). This leads to the emergence of spinal reflex mechanisms that promote uninhibited detrusor contractions during urine storage (a condition known as detrusor overactivity, DO) and further DSD. DSD can be particularly dangerous: as the bladder contracts against a closed EUS, it generates increased pressures, which can lead to renal injury and loss of bladder compliance (Kaplan et al., 1991).

Current therapy for LUT dysfunction after SCI focuses on managing these complications without addressing the underlying cause or attempting to normalize or restore bladder function. Historically, a variety of electrical stimulation techniques have been proposed to improve urine storage by increasing capacity and reducing DO; and improve urine voiding by stimulating detrusor contraction and sphincter relaxation at patient-determined intervals. These strategies include direct electrical stimulation of the pelvic nerve (Holmquist, 1968), the sacral nerve (Sievert et al., 2010; Granger et al., 2013) or the pelvic plexus and bladder wall (Walter et al., 2005). While promising, these approaches have not been widely implemented due to their invasive nature and need for concurrent sensory denervation (e.g., the sacral nerve stimulator) or lack of long-term effectiveness (e.g., bladder wall plexus stimulator). Additionally, these approaches act locally to reduce DO or induce detrusor contraction by direct nerve stimulation. On the other hand, neuromodulating approaches, such as the one presented here, may promote restoration of LUT function by activating the inherent to the spinal neural networks and re-establish communication between neural centers separated by an injury.

Epidural spinal cord stimulation (ES) has been previously introduced as a novel approach to activate neural networks and enable a variety of functions after SCI (Gerasimenko et al., 2008). This approach has been demonstrated to enable volitional motor function in animal models and humans after SCI (Courtine et al., 2009; Harkema et al., 2011). Further, ES of the lumbosacral spinal cord has shown potential utility for activating neural networks associated with LUT function in rodents (Gad et al., 2014; Abud et al., 2015). However, ES is highly invasive, which limits its application and scope. Recently, transcutaneous spinal cord stimulation (TSCS) has been developed as a non-invasive method to activate neural circuits in the human spinal cord in order to enable function of upper (Gad et al., 2018a; Inanici et al., 2018), trunk (Rath et al., 2018), and lower extremity (Gerasimenko et al., 2015; Gad et al., 2017). We have demonstrated the feasibility and utility of using TSCS over the thoracolumbar spine to activate the detrusor in neurologically intact rhesus macaques (Grahn et al., 2017; Gad et al., 2018b). In this report, we demonstrate that Transcutaneous Electrical Spinal Stimulation for LUT functional Augmentation (TESSLA) can activate and improve LUT function after a severe SCI. We hypothesize that TESSLA activates the spinal neural networks that are active in controlling LUT function. Key features of TESSLA include its non-invasiveness and its subject specific adaptability for the selection of stimulation sites and parameters. In addition, the non-invasive feature of TESSLA may be appealing to a broader population of subjects at a modest cost compared to surgically invasive neuromodulatory devices.

## Methods

This study was approved by the Institutional Review Board of Rancho Research Institute, the research arm of Rancho Los Amigos National Rehabilitation Center, Downey, CA. All research participants signed an informed consent form before the start of the study and consented to their data being used in future publications and presentations. Seven individuals (Four male and three females) with SCI at T11 or above who used clean intermittent catheterization to manage the LUT were recruited. Each subject had a stable SCI that occurred at least one year prior to study initiation. Baseline demographic, medical, and urodynamic parameters of study participants are shown in Table 1. The experiments were carried out with use of a proprietary non-invasive Transcutaneous Electrical Spinal Cord Stimulator (NeuroRecovery Technologies, Inc.). Six participants were tested for a 6-h period over 2 days (3 h per day), and one participant underwent additional testing on Day 3. On Day 1, the LUT was mapped to spinally evoked responses. Spinal stimulation was delivered at 0.5 Hz (individually at T11 and L1) with current starting at 10 mA and increasing at increments of 10–200 mA (or until tolerable or responses had plateaued). On Day 2, a baseline urodynamic procedure without stimulation was performed with the subjects either in seated (*n* = 4) or supine (*n* = 3) position. Next, TESSLA was delivered at 30 Hz at T11. Urodynamic recording was commenced and the bladder was filled until a detrusor contraction was elicited. The stimulation was turned off and the bladder was fully emptied. Subsequently, the bladder was filled to a 75% capacity. To ensure that reflex bladder contraction did not occur, detrusor pressure was monitored for at least 2 min. Next, TESSLA was delivered at 1 Hz at T11 to initiate voiding. When voiding was completed, the bladder was emptied completely. On Day 3, one subject (566729), who was able to sit comfortably on a modified padded toilet seat was asked to undergo a uroflow test in the absence and presence of TESSLA.

**Table 1:** Baseline demographic and medical characteristics and urodynamic parameters of study participants.

**Demographic and Medical Characteristics**	**566729**		**399343**		**955941**		**535144**		**25801**		**151947**		**573487**	
Gender	Male		Female		Female		Female		Male		Male		Male	
Age (years)	32		49		43		32		48		38		28	
Years since injury	1.25		5.67		3.08		2.08		1.00		23		3.56	
Level of injury	T4		C6		T11		T6		C3		T6		T8	
ASIA classification	A		B		C		A		C		A		A	
Bladder Management	CIC		CIC		CIC		CIC		CIC		CIC		CIC	
LUT Sensation	No		Yes		Yes		Yes		No		Yes		Yes	
Urinary incontinence	One episode per day		None		Rare		One episode per week		Multiple times a day		None		Few episodes per week	
LUT medications	None		Fesoterodine 8 mg Daily		Oxybutynin ER 15 mg Daily		Mirabegron 50 mg Daily		None		Onabotinum toxin		None	
Bowel regimen	Sup/DS		Sup/DS		Enema/DS		DS		DS		Sup/DS		DS	
Autonomic dysreflexia	No		Yes		No		Yes		Yes		No		No	
LUT Characteristics	TESSLA		TESSLA		TESSLA		TESSLA		TESSLA		TESSLA		TESSLA	
	Off	On	Off	On	Off	On	Off	On	Off	On	Off	On	Off	On
Vol at first detrusor contraction (ml)	205	281	164	160	356	500	71	212	87	198	258	323	52	95
Voiding efficiency	12.2%	39.8%	8.5%	36.2%	0.0%	10.5%	64.7%	100%	0.0%	54.4%	7.3%	14.8%	96.2%	100%
Post-void residual (ml)	180	127	150	90	440	335	25	0	160	155	330	350	2	0
Sensation of fullness	No	No	No	No	No	No	No	No	Yes	Yes	Yes	Yes	No	Yes
Sensation of detrusor contraction	No	No	No	Yes	No	Yes	No	No	No	Yes	No	No	No	No
Detrusor-sphincter dyssenergia	Yes	No	Yes	No	Yes	No	Yes	No	Yes	No	Yes	No	Yes	No
Maximum change in Pdet during voiding (cm of H20)	36	65	45	45	NA	31	113	90	NA	51	34	20.7	114	56
Maximum change in Pura during voiding (cm of H20)	92	−44	−2	11	NA	−30	40	−15	NA	7	8.2	−11	77	−53

*LUT, Lower Urinary Tract; CIC, Clean intermittent catheterization, Sup, Suppository; DS, Digital Stimulation*.

*Note the green boxes identify parameters that demonstrate a positive change with TESSLA On compared to TESSLA Off, whereas the red boxes identify parameters that demonstrate a negative change*.

## Urodynamic study

The external genitalia were prepared and draped in sterile fashion. The urethra was lubricated and a 7-Fr triple lumen T-DOC® Air-Charged™ urodynamic catheter (Laborie, Ontario, Canada) was placed per urethra with the distal pressure port positioned within the bladder and the proximal pressure port within the EUS. Position within the urethral sphincter was confirmed by the pressure reading from the proximal port. Electromyography (EMG) of the EUS was performed with bilateral patch electrodes placed at the anal verge. Abdominal pressure measurement was obtained via a 7-Fr single-lumen T-DOC® Air-Charged™ catheter (Laborie, Ontario, Canada) placed per rectum. Urodynamic studies (UDS) were performed according to International Continence Society urodynamic standards using a Goby™ urodynamic system (Laborie, Ontario, Canada) with a fill rate of 30 ml/min (Rosier et al., 2016). In addition to EMG, the following urodynamic parameters were recorded: vesical pressure (P_ves_), abdominal pressure (P_abd_), EUS pressure (P_ura_), and flow rate. Detrusor pressure (P_det_) was obtained by subtracting P_abd_ from P_ves_. Closure pressure (P_clo)_ was obtained by subtracting P_ves_ and P_ura_. Bladder capacity was defined as the bladder volume at which urinary incontinence occurred. Post-void residual saline was withdrawn by using the indwelling urodynamic catheter to empty the bladder following urination. Voiding efficiency was defined as

$$\begin{array}{l} \frac{Voided\;Volume}{Voided\;Volume+Postvoid\;Residual}.\;. \end{array}$$

## Non-invasive spinal cord stimulation

TSCS was delivered between spinous processes using 2.0 cm-diameter round gel adhesive electrodes (Axelgaard, ValuTrode® Cloth) as cathode and two 5.0 × 10.0 cm^2^ rectangular electrodes (Axelgaard, ValuTrode® Cloth) placed over the iliac crests as anode.

### Mapping

TSCS was delivered individually along the midline between spinous processes at T11-T12, and L1-L2 at 0.5 Hz to map the LUT. Stimulation was started at 10 mA and increased in increments of 10–200 mA or until it was no longer tolerable. Each intensity of stimulation determined by the pulse width was repeated 5 times to assess reproducibility of responses.

### Testing

**Figure 1:**
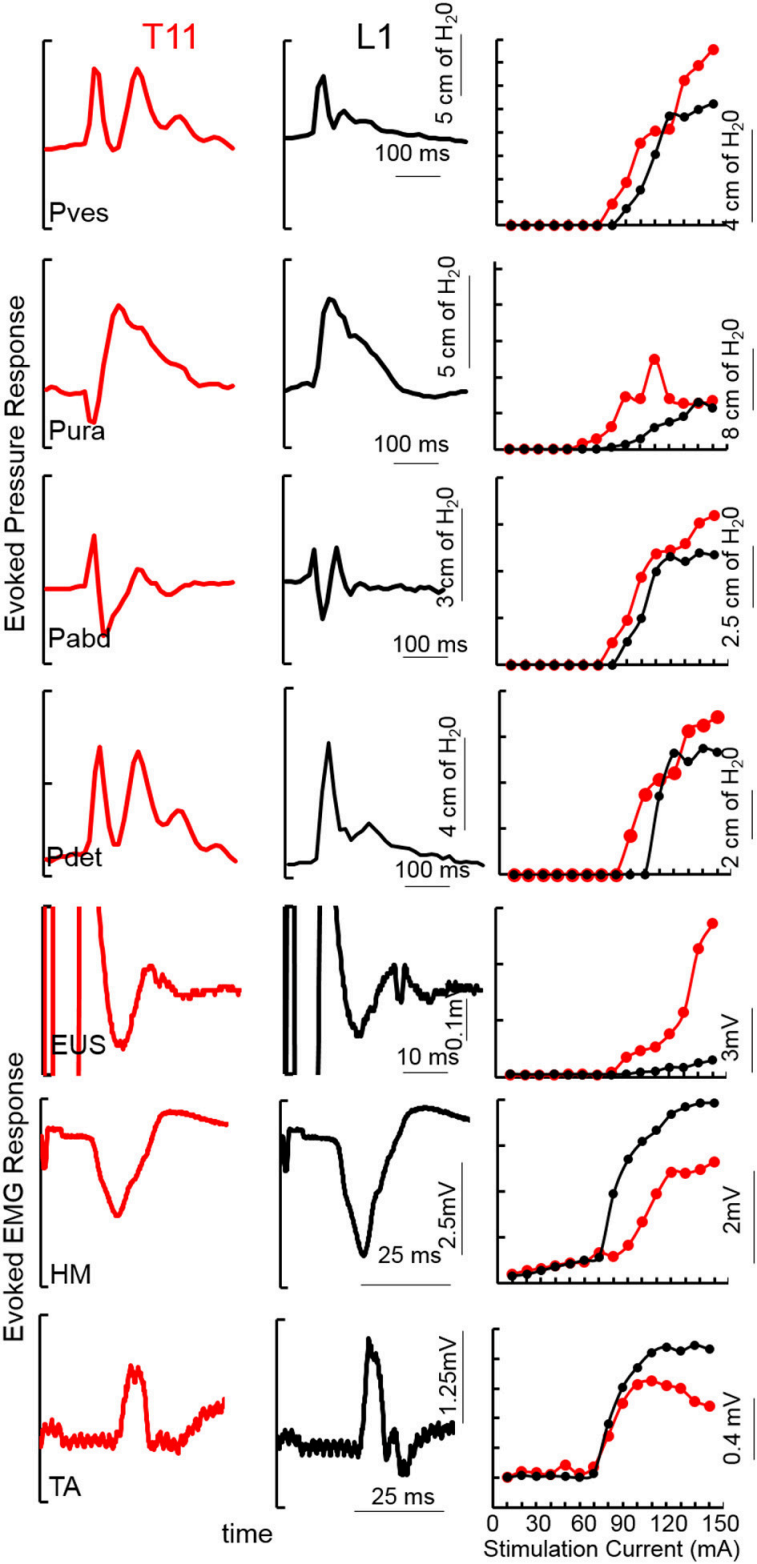
An example of TSCS evoked pressure changes and EMG responses (average of 5 responses) from the vesicular (Pves), urethral (Pura), abdominal (Pabd) and detrusor (Pdet) pressures and Urethral sphincter (EUS) EUS, Hamstring (HM) and Tibialis Anterior (TA) EMGs at 150 mA TSCS between T11-T12 and L1-L2 vertebral processes from a subject (566729, AIS A, T4). Recruitment curves for the example showed on the left.

The stimulation intensity and site chosen were based on the recruitment curves obtained from mapping studies (Figure 1). Similar methods have been used in our animal studies (Gad et al., 2014, 2016). During functional UDS studies with TESSLA, the frequency was set at either 1 or 30 Hz (see main text). The intensity used during 1 and 30 Hz TESSLA was determined based on responses observed during the mapping studies.

## Urodynamic studies with TESSLA

### 30 Hz stimulation

The bladder was emptied via the indwelling urodynamic catheter. TESSLA at 30 Hz was initiated at T11. The bladder was then infused with saline at a rate of 30 ml/min until a detrusor contraction occurred. At the end of the detrusor contraction, the bladder was manually emptied via the infusion port to calculate the residual volume.

### 1 Hz stimulation

The bladder was emptied via the indwelling urodynamic catheter. The bladder was filled with saline to 75% of bladder capacity (determined by baseline UDS) at a rate of 30 ml/min. Filling was then stopped and detrusor pressure was monitored for at least 1–2 min to ensure that no detrusor contraction occurred. Next, TESSLA was delivered at a frequency of 1 Hz at T11. Within 60 s of stimulation, a detrusor contraction was induced. After the induced detrusor contraction ended, bladder was manually emptied via the infusion port to calculate the residual volume.

## Data analysis

The studies provided several urodynamic parameters were analyzed including: (1) infused volume; (2) post void residual; (3) voiding efficiency; (4) peak pressure during detrusor and urethral contraction; (5) peak-to- peak pressures and EMG amplitude during mapping studies. These average peak to peak responses for each intensity were used to generate the recruitment curves for each site of stimulation. (6) Co-activation between Pdet and Pura per second during voiding was defined as $\frac{Pdet^{*}Pura}{voiding\;duration\;}$to assess the level of DSD, (7) Bladder capacity was defined as the volume at which first DO with leakage occurred. (8) Infused volume at which first DO occurred was used to quantify severity of DO.

## Statistical analysis

The paired t test was used to compare data between groups with TESSLA Off and TESSLA On using Graphpad software.

## Results

Mapping over T11-12 demonstrated detrusor contractions with lower levels of activation of the urethra and abdomen, whereas stimulation of L1-2 minimally activated the detrusor, urethra and abdomen (Figure 1). Note the unique pattern of responses in the Pves, Pura, Pabd, and Pdet. Lower extremity responses were consistent with those reported earlier (Sayenko et al., 2015a; Gad et al., 2017). The contractions in the detrusor and urethra, identified via pressure changes, were used as indicators to identify appropriate sites for functional studies. The site at which the largest detrusor contraction was observed at the lowest stimulation intensity was used for functional studies in the next phase.

**Figure 2:**
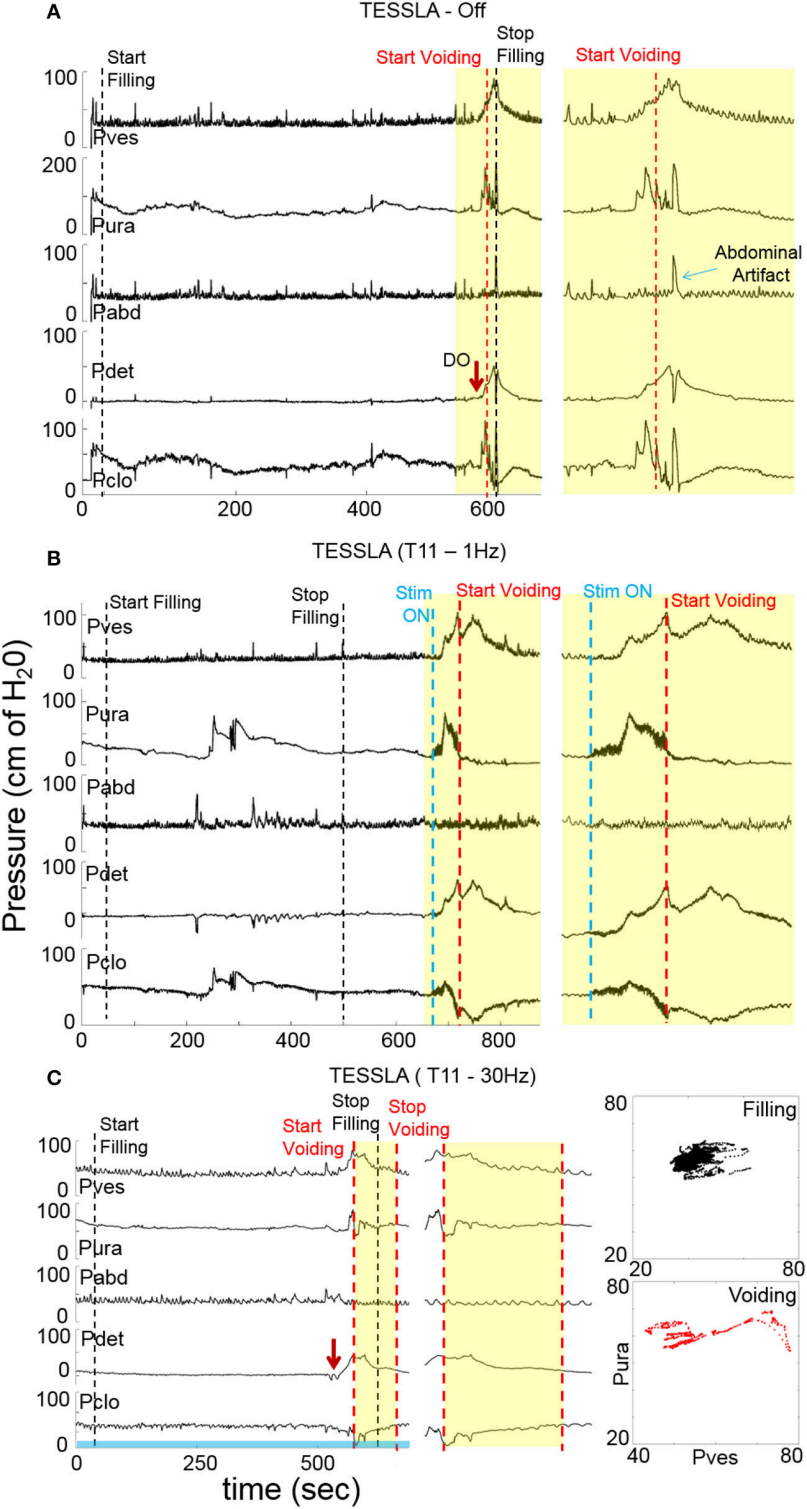
Examples of urodynamic recording from a representative subject (566729) **(A)** without TESSLA, with **(B)** TESSLA at 1 Hz (T11) and with **(C)** TESSLA at 30 Hz (T11). Note the presence of detrusor over activity (DO) with 205 ml infused (Table 1) and high level of detrusor sphincter dyssynergia (DSD) in the absence of TESSLA but a greater level of reciprocal activation between the Pura and Pdet in the presence of TESSLA and during voiding. Note the increased bladder capacity (281 ml, Table 1) in the presence of TESSLA at 30 Hz compared to baseline as well as the reduced DSD during voiding. Pdet is defined as Pves-Pabd and Pclo is defined as Pves-Pura.

**Figure 3:**
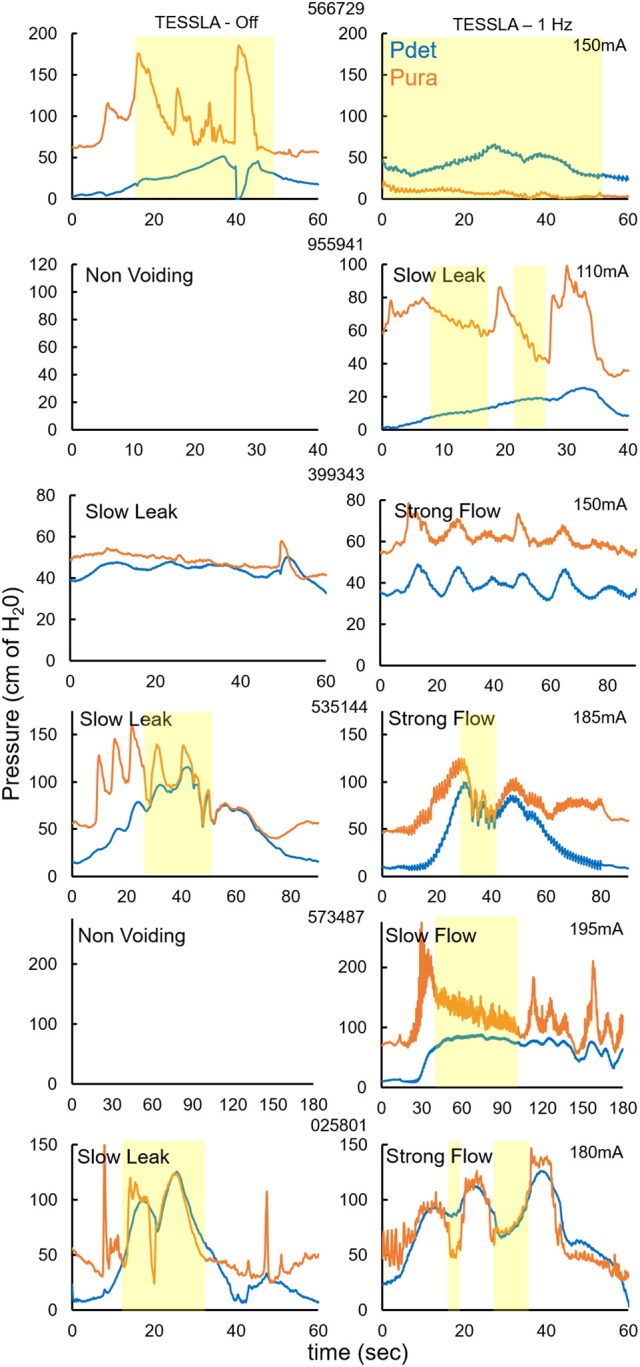
Pdet and Pura during voiding without and with TESSLA (1 Hz) for the 6 individual subjects. Yellow highlight identifies the region of voiding. Note: 2 subjects (955941, 573487) demonstrated a non-voiding responses during TESSLA Off, thus the pressure traces are not included.

**Figure 4:**
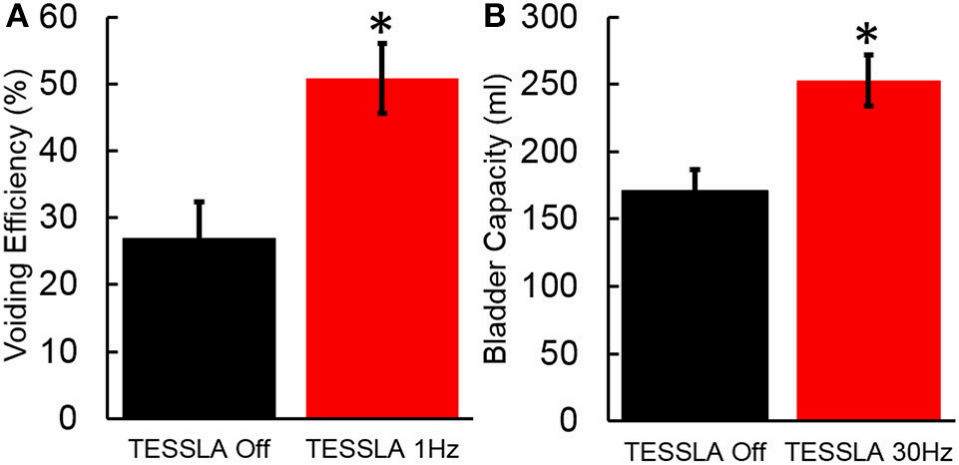
**(A)** Voiding efficiency for the 7 individuals tested in the absence of TESSLA and TESSLA at 1 Hz. **(B)** Bladder capacity in the absence of TESSLA and TESSLA at 30 Hz. ^*^ significantly different from TESSLA Off at *P* < 0.05 (Statistical difference identified via paired *t*-test).

**Figure 5:**
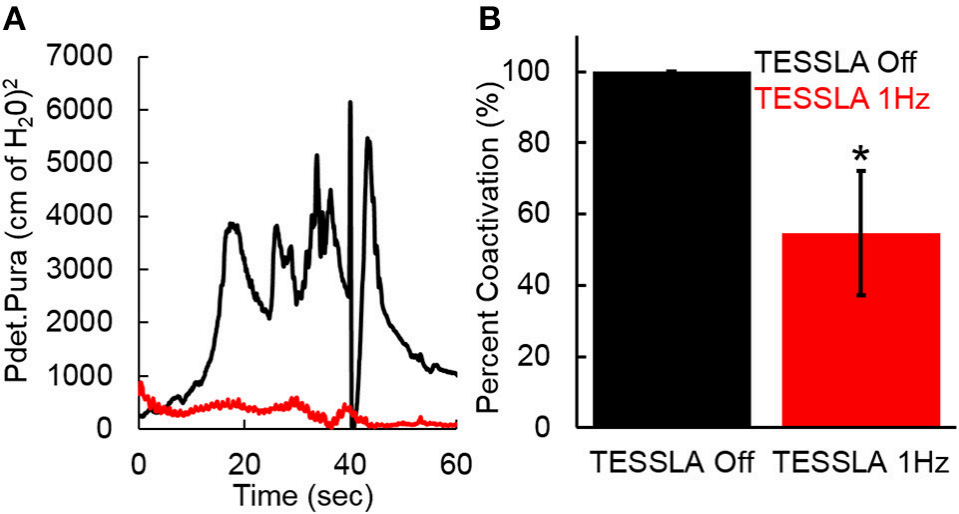
**(A)** Co-activation between Pdet and Pura during voiding with TESSLA off (black) and TESSLA at 1 Hz (red) in a single subject (566729). **(B)** Normalized (to TESSLA Off) co-activation between Pdet and Pura per second during voiding for the subjects (*n* = 5) that demonstrated voiding with TESSLA off. ^*^ significantly different from TESSLA Off, demonstrating lowered level of DSD with TESSLA 1 Hz compared to TESSLA Off (Statistical difference identified via paired *t*-test).

Baseline UDS demonstrated features that are typical of UDS recordings in individuals with SCI (Figure 2A, Table 1; Weld and Dmochowski, 2000). TESSLA delivered at T11 at 1 Hz resulted in improved voiding efficiency (VE), increased flow rate, decreased residual volume and improved coordination between the detrusor and sphincter (Figures 2B, 3, Supplementary Movie). The voiding efficiency increased from 26.99 ± 15.41 to 50.80 ± 5.25 % (*P* < 0.05, *n* = 7). In contrast, TESSLA delivered at 30 Hz at T11 resulted in reduced DO during urine storage, i.e., increased bladder capacity (Figures 2C,4) and improved detrusor-sphincter coordination during voiding (Figures 3, 5, *n* = 5, *P* < 0.05). The bladder capacity increased from 170.54 ± 15.86 to 252.59 ± 18.91 ml (*P* < 0.05, *n* = 7). Since the SCI subjects were unable to generate a voluntarily induced detrusor contraction, the infused volume at which an involuntary contraction in the detrusor was used as a surrogate biomarker to assess the severity of DO (brown arrow, Figure 2). As shown in Figure 4, significantly decreased DO was observed when TESSLA at 30 Hz was applied. When UDS was repeated without stimulation, reversal to baseline was observed. Stimulation at multiple sites (T11+L1+Co1) used during locomotor training in the past (Gerasimenko et al., 2015; Sayenko et al., 2015b) did not enable efficient bladder voiding.

**Figure 6:**
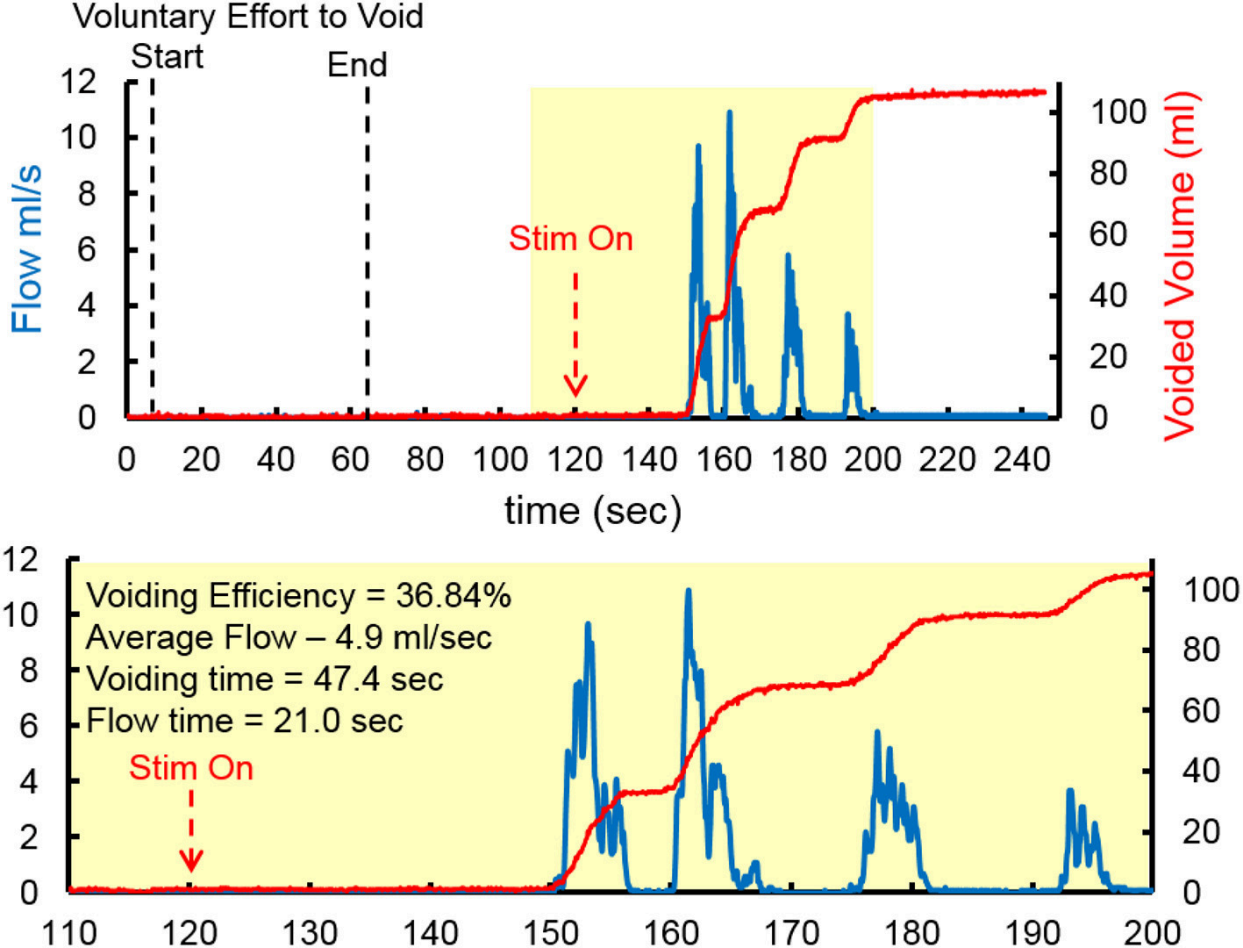
Representative Uroflow conducted on one subject in the absence and presence of TESSLA (150 mA, 1 Hz at T11). Note the start of voiding and flow only after TESSLA is turned on (Supplementary movie).

At the end of the study, one subject underwent an unintubated uroflow test. The subject was initially asked to void in the absence of TESSLA and subsequently in the presence of TESSLA. A voiding contraction was induced when TESSLA was turned on at T11 at 1 Hz with a VE of 36.84% and average flow rate of 4.9 ml/s (Figure 6, Supplementary movie). The procedures were well tolerated by all subjects with no change in blood pressure, heart rate, skin irritation (during and after the procedures) and change in spasticity (after the procedure). None of the subjects reported episodes of increased incontinence during the days following the experimental sessions. Even though urodynamic procedures are known to trigger episodes of autonomic dysreflexia (AD) (Giannantoni et al., 1998) due to overfilling of the bladder, care was taken to ensure that the bladder was not overfilled and did not induce symptoms of AD.

## Discussion

Previous studies have shown feasibility of using invasive ES to selectively activate neuronal networks in experimental models. ES over the L3 spinal segments activated the spinal EUS bursting center in rats and promoted the switch between the bladder storage automaticity phase to voiding with improved coordination between detrusor and sphincter in both intact and spinal animals (Abud et al., 2015). Previous studies have also shown that LUT function improves with ES aimed at restoring locomotor function after SCI (Harkema et al., 2011; Gad et al., 2014), including improvement in continence, bowel, and sexual function in human subjects (Hubscher et al., 2018). While locomotor-based therapies resulted in improvements in bladder capacity and voiding efficiency, multiple studies suggest that fine tuning of stimulation parameters play an important role in defining the level of functional recovery after SCI (Gad et al., 2013; Rejc et al., 2017). Each of these above-mentioned studies highlight the automaticity that is intrinsic to the spinal networks controlling LUT function and the ability to activate the appropriate neural networks (spinal micturition centers) based on the site and pattern of stimulation. Continuously delivered TESSLA with a partially filled bladder results in an overall increase in detrusor pressure past a threshold while lowering the urethral pressure to initiate voiding. This is one of the key features of the TESSLA induced voiding, wherein TESSLA activates the neural networks that control LUT function to initiate voiding. Multisite stimulation that has been reported to be most effective in enabling locomotion [T11+L1+Co1, (Gerasimenko et al., 2015; Sayenko et al., 2015b)] were not effective in enabling voiding. Further, it was necessary to fine-tune stimulation parameters for each subject. Though 30 Hz stimulation resulted in increased bladder capacity (reduced DO) and improved coordination between detrusor and sphincter (reduced DSD), the overall voiding efficiency was similar to baseline, suggesting the need to fine tune the spinal circuitry based on the intended application i.e., low frequency to initiate voiding (Gad et al., 2014) vs. high frequency to increase bladder capacity (present data) and to enable locomotor function (Lavrov et al., 2006; Gad et al., 2016).

Modeling (Danner et al., 2011) and experimental (Hofstoetter et al., 2018) studies have shown that transcutaneous stimulation (Sayenko et al., 2015a) can depolarize at least a subset of the same neural structures as recruited by implanted epidural electrodes (Sayenko et al., 2014). Data obtained from experiments with epidural stimulation and transcutaneous stimulation demonstrate that, with increasing intensity, the stimulus response relationship of the early (short latency) and medium components in many muscles shares some characteristics with the H-reflex and M-wave interactions and can recruit dorsal afferents, interneurons, and motor neurons. The present study shows that TESSLA can be used to stimulate the neural circuitries in the spinal cord of human subjects with SCI and facilitate LUT function by reducing DO, decreasing DSD, increasing bladder capacity and enabling voiding. Each of these features has significant clinical and functional implications. Controlling DO and increased bladder capacity lead to fewer incontinence episodes, thus benefitting patients' health and self-confidence. Decreasing DSD lowers the risks of high pressure voiding, loss of bladder compliance, and kidney injury. Additionally, our finding that TESSLA mediates recovery of bladder-sphincter synergy suggests that coordination between the detrusor and the EUS can occur at the spinal level, thus challenging the dogma that bladder-sphincter coordination is facilitated solely by the brainstem. TESSLA may effect these changes by reducing pathological spinal mechanisms that arise after a SCI, e.g., the emergence of spinal reflex mechanisms mediated by unmyelinated vesical afferents (C-fibers) (de Groat and Yoshimura, 2010). Finally, TESSLA directly addresses one of the primary dysfunctions caused by SCI, i. e., the inability to void on command.

Several neuromodulation systems have been previously developed to improve LUT function after SCI (Gaunt and Prochazka, 2006). Examples of such systems include the dorsal penile/clitoral nerve, tibial nerve, and sacral nerve stimulators. Of these, the dorsal penile nerve stimulator has been most extensively studied in the SCI population. This device takes advantage of a urethral guarding reflex: by stimulating a branch of the pudendal nerve (in this case, the dorsal penile nerve), detrusor contractions are inhibited. Dorsal penile nerve stimulation has been assessed in several studies of SCI patients with promising results for decreasing incontinence and promoting a larger bladder capacity (Kirkham et al., 2001; Lee et al., 2003). Despite these successes, the penile nerve stimulator has several limitations, including the need for continuous stimulation to inhibit contractions, and the practical implications of attaching the device to the genitalia. Furthermore, in contrast to TESSLA, dorsal penile nerve stimulation does not address recovery of bladder sensation or promote bladder emptying. Indeed, we are not aware of any neuromodulation techniques that stimulate the spinal cord directly in a non-invasive manner to facilitate LUT functional recovery after SCI.

Each of the improvements in LUT function observed with TESSLA is consistent with the priorities set by NIBIB/NIH at the workshop entitled “Addressing Paralysis through Spinal Stimulation Technologies” in November, 2014 (Pettigrew et al., 2017). The data presented here directly addresses and validates five out of the six objectives proposed for restoring bladder function. In addition, we hypothesize that TSCS could address other pelvic autonomic functions such as bladder, bowel and sexual function, discussed at the consortium. While the discussion at the consortium focused on epidural stimulation, we would like to emphasize that TESSLA offers the additional unique feature of being non-invasive.

In conclusion, TESSLA offers several advantages over current therapies for LUT dysfunction due to SCI. First, TESSLA is non-invasive. If the intervention is not tolerated by the subject, it can be immediately discontinued. Second, we demonstrate that TESSLA can modulate the spinal networks devoted to micturition with a critical level of specificity. Third, TSCS and training used to enable upper (Gad et al., 2018b) and lower extremity (Gerasimenko et al., 2015) function led to plastic changes in the spinal networks in absence of stimulation. Similarly, TESSLA may result in plastic changes to the neural circuitry controlling bladder filing, voiding, and sensation. However, further studies are needed to determine impact of TESSLA and training on LUT function in the longterm.

## New & noteworthy

TESSLA leads to improvement and normalization of the lower urinary tract function after spinal cord injury. TESSLA offers several advantages over current therapies for LUT dysfunction due to SCI. First, it is non-invasive. If the intervention is not tolerated by the subject, it can be immediately discontinued. Second, we demonstrate that TESSLA can modulate the activity of the spinal cord to elicit a specific response in the lower urinary tract. Third, TESSLA can be easily integrated with other rehab programs that a patient may be undergoing.

## Author contributions

PG, VE, and EK designed the study. PG, EK, HZ, and KL performed the experiments. PG analyzed the data. PG, VE, and EK wrote the initial manuscript. PG, VE, KL, HZ, and EK edited a final version of the manuscript.

### Conflict of interest statement

PG and VE, researchers on the study team hold shareholder interest in NeuroRecovery Technologies and hold certain inventorship rights on intellectual property licensed by The Regents of the University of California to NeuroRecovery Technologies and its subsidiaries. The remaining authors declare that the research was conducted in the absence of any commercial or financial relationships that could be construed as a potential conflict of interest.

## Supplementary material

Supplementary Movie Video demonstrating a uroflow recording without TESSLA and with TESSLA at 1 Hz and animation and 3D representation demonstrating the coordination between the detrusor, urethral sphincter, and flow.
